# Crystal structure of *Staphylococcus aureus* lipase complex with unsaturated petroselinic acid

**DOI:** 10.1002/2211-5463.13808

**Published:** 2024-05-16

**Authors:** Julia Kitadokoro, Shigeki Kamitani, Yukiko Okuno, Takaaki Hikima, Masaki Yamamoto, Takatsugu Hirokawa, Kengo Kitadokoro

**Affiliations:** ^1^ Faculty of Molecular Chemistry and Engineering, Graduate School of Science and Technology Kyoto Institute of Technology Japan; ^2^ Department of Nutrition, Graduate School of Human Life and Ecology Osaka Metropolitan University Habikino Japan; ^3^ Medical Research Support Center, Graduate School of Medicine Kyoto University Japan; ^4^ SR Life Science Instrumentation Team, Life Science Research Infrastructure Group, Advanced Photon Technology Division RIKEN SPring‐8 Center Sayo‐gun Japan; ^5^ Transborder Medical Research Center University of Tsukuba Japan; ^6^ Division of Biomedical Science, Faculty of Medicine University of Tsukuba Japan

**Keywords:** anti‐obesity, crystal structure, inhibitor complex, pathogenic enzyme, *Staphylococcus aureus*, unsaturated fatty acid

## Abstract

*Staphylococcus aureus* produces large amounts of toxins and virulence factors. In patients with underlying diseases or compromised immune systems, this bacterium can lead to severe infections and potentially death. In this study, the crystal structure of the complex of *S. aureus* lipase (SAL), which is involved in the growth of this bacterium, with petroselinic acid (PSA), an inhibitor of unsaturated fatty acids, was determined by X‐ray crystallography. Recently, PSA was shown to inhibit *S. aureus* biofilm formation and the enzymatic activity of SAL. To further characterize the inhibitory mechanism, we determined the half‐inhibitory concentration of SAL by PSA and the crystal structure of the complex. The IC_50_ of the inhibitory effect of PSA on SAL was 3.4 μm. SAL and PSA inhibitors were co‐crystallized, and diffraction data sets were collected to 2.19 Å resolution at SPring‐8 to determine the crystal structure and elucidate the detailed structural interactions. The results show that the fatty acid moiety of PSA is tightly bound to a hydrophobic pocket extending in two directions around the catalytic residue Ser116. Ser116 was also covalently bonded to the carbon of the unsaturated fatty acid moiety, and an oxyanion hole in SAL stabilized the electrons of the double bond. The difference in inhibitory activity between PSA and ester compounds revealed a structure–activity relationship between SAL and PSA. Additional research is required to further characterize the clinical potential of PSA.

## Graphical abstract

*Staphylococcus aureus* lipase (SAL) is a glycerol ester hydrolase involved in the growth of this bacterium and petroselinic acid (PSA) has been shown to inhibit SAL. In this study, we determined the IC50 of PSA for SAL and the crystal structure of the SAL‐PSA complex. Further research is required to uncover the clinical potential of PSA.

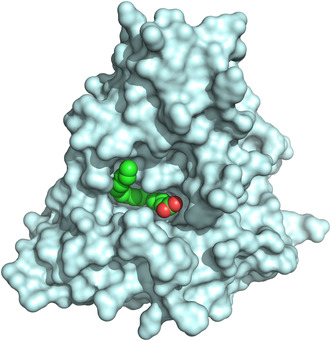

**Abbreviations**
DGL: Dog gastric lipase
Geh: glycerol ester hydrolase
HGL: Human gastric lipase
MRSA: Methicillin‐resistant *S. aureus*
OA: oleic acid
*p*NPB: *p*‐nitrophenyl butyrate
PSA: petroselinic acid
PSM: petroselinic acid methyl ester
SAL: *Staphylococcus aureus* lipase
SAL‐PSA: *Staphylococcus aureus* lipase PSA complex
TLL: *Thermomyces lanuginose* lipase

*Staphylococcus aureus* is an anaerobic gram‐positive cocci, a grape‐shaped bacterium found in human nasal passages, purulent wounds, and skin surfaces. It is abundant in the crusts of wounds, enters the body through wounds, produces many virulence factors, and causes various diseases [1, 2]. When the elderly, immunocompromised persons after surgery, and infants become infected, it can lead to severe infections such as pneumonia and sepsis [3]. Atopic dermatitis occurs when there are high levels of *S. aureus* on the skin surface [4]. It produces many types of toxin proteins and virulence factor enzymes that can cause pneumonia, peritonitis, sepsis, meningitis, food poisoning, toxic shock syndrome, and enteritis [5, 6]. Methicillin‐resistant *S. aureus* (MRSA), which is resistant to antibiotics, is also a cause of nosocomial and community‐acquired infections [7, 8]. It is known to form aggregates called biofilms on material surfaces, and biofilm infections that occur on the surfaces of medical devices such as catheters, pacemakers, and artificial joints are major clinical problems [9, 10].

*Staphylococcus aureus* lipase (hereafter abbreviated as SAL, EC.3.1.1.3) has potent cytotoxic activity against certain types of cells and may be involved in the etiology of *S. aureus* disease [11]. Excessive amounts of SAL have been found in many patients who died after infection with MRSA [11]. SAL is also involved in the growth of *S. aureus*, and inhibitors of this enzyme are thought to inhibit the growth of this bacterium [12].

*Staphylococcus aureus* lipase is secreted as an enzyme consisting of 394 amino acid residues with a molecular weight of 45.7 kDa. It belongs to the glycerol ester hydrolase (Geh) family and is the enzyme that degrades triacylglycerols. SAL is classified as a typical serine protease‐like α/β‐hydrolase with a catalytic triad consisting of residues Ser116, Asp307, and His349 residues [13]. It also has an oxyanion hole consisting of nitrogen atoms in the main chain of Phe17 and Met71, which maintain a stable structure during the formation of reaction intermediates [13].

The antimicrobial activity of long‐chain alkyl fatty acids on microorganisms has been demonstrated previously [14, 15]. Although the mechanism is still unclear, it is known that long‐chain alkyl fatty acids disrupt biofilms formed by bacteria, suggesting that antimicrobial action occurs by inhibiting biofilm formation [14, 15]. Recently, a group at Yeungnam University discovered that cis‐petroselinic acid (hereafter referred to as PSA, the structural formula is shown in Fig. 1), a regioselective isomer of oleic acid, one of the long‐chain alkyl fatty acids found in parsley oil, inhibits biofilm formation by *S. aureus*, and also has inhibitory activity against SAL [16].

**Fig. 1:**
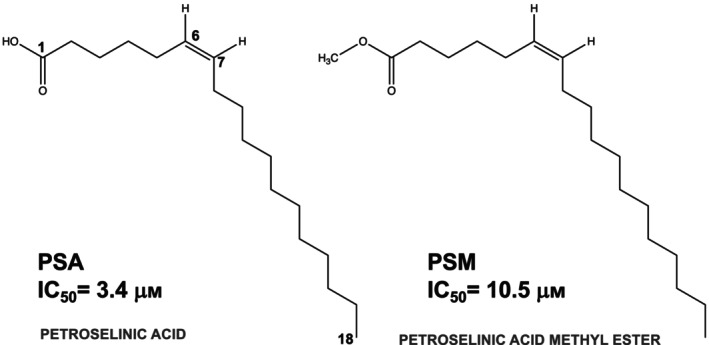
Chemical structures of PSA and PSM, with IC values. Relative activities were measured at different inhibitor concentrations. Both compounds form a double bond between the C6 and C7 positions.

In this paper, we determined the half‐inhibitory concentration of SAL by PSA and the crystal structure of the complex. We also elucidated the structure–activity relationship between SAL and PSA by comparing the IC_50_ of PSA with its ester compound.

## Results and Discussion

### Results of IC_50_ half‐inhibition values for PSA and PSM

**Fig. 2:**
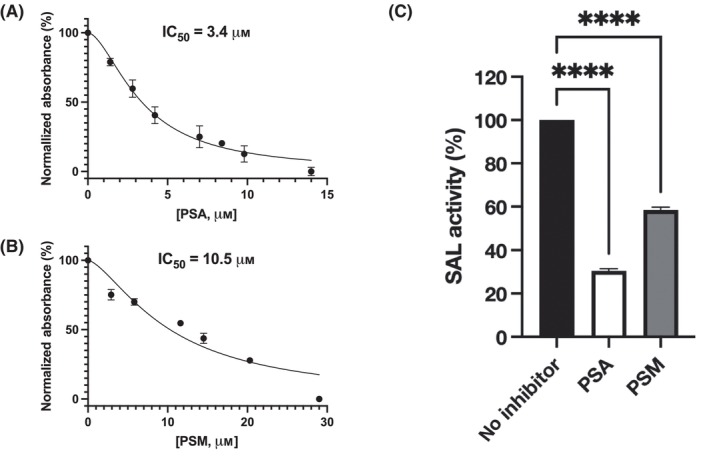
Inhibition of SAL by PSA and PSM. The IC_50_ value was determined using the IC_50_ calculator (https://www.aatbio.com/tools/ic50‐calculator). SAL activity towards *p*‐nitrophenylbutyrate. Enzymatic activity was measured by monitoring *p*‐nitrophenol production Each data point is presented as mean ± SEM with error bars representing SEM (*n* = 3). (A) The IC_50_ of PSA versus SAL. (B) The IC_50_ of PSM versus SAL. (C) Comparison of increased SAL activity of the same concentrations of PSA and PSM (14 μm each). Values without inhibitors are taken as 100%. The statistical software used was prism9 (GraphPad, Boston, MA, USA), with one‐way ANOVA tests and Dunnet for multiple comparisons.

The activity of PSA and its methyl ester form, PSM, were measured using a spectrophotometer. The IC_50_ half‐inhibitory concentration of PSA was 3.4 μm, and that of PSM was 10.5 μm, indicating that PSA inhibited SAL approximately three‐fold more strongly than PSM (Figs 1 and 2).

### Crystallization, X‐ray structure analysis of the SAL in complex with unsaturated fatty acids

The SAL complex with various unsaturated fatty acids was crystallized in the same manner as previously reported [13], yielding large diamond‐shaped crystals in about 2 weeks (Fig. S1). SAL‐PSA complex was crystallized at 295 K in the presence of 2.45 m sodium formate in 100 mm acetate (pH 4.5). Diffraction data sets of SAL/PSA complex crystals were measured and analyzed at the BL44XU beamline at SPring‐8 at 2.19 Å resolution (Table 1). The space group was *P*4_1_22, cell constants *a* = *b* = 132.3, *c* = 248.6 Å, and α, β, γ = 90°, and it was nearly isomorphic with the native crystal, containing two molecules in an asymmetric unit.

**Table 1:** Data collection, processing, and refinement. Outer shell values are shown in parentheses. Completeness for all reflections and the highest resolution shell in parentheses.

	SAL/PSA complex	S116A/PSA complex	SAL/PSA covalent complex
PDB ID	8K7P	8K7Q	8YIB
Diffraction source	BL44XU/SPring‐8	BL44XU/SPring‐8	BL44XU/SPring‐8
Wavelength (Å)	0.90	0.90	0.90
Temperature (K)	100	100	100
Detector	Eiger X 16M	Eiger X 16M	Eiger X 16M
Crystal–detector distance (mm)	300	300	300
Rotation range per image (°)	0.1	0.1	0.1
Total rotation range (°)	180	180	180
Exposure time per image (s)	0.1	0.1	0.1
Space group	*P*4_1_22	*P*4_1_22	*P*4_1_22
*a, b, c* (Å)	132.3, 132.3, 248.6	131.4, 131.4, 249.9	132.6, 132.6, 249.8
α, β, γ (°)	90.0, 90.0, 90.0	90.0, 90.0, 90.0	90.0, 90.0, 90.0
Mosaicity (°)	0.049	0.127	0.220
Resolution range (Å)	50–2.19 (2.32–2.19)	50–2.02 (2.14–2.02)	50–2.27 (2.41–2.27)
Total no. of reflections	1 546 350	1 932 995	1 380 373
No. of unique reflections	217 650	272 631	195 132
Completeness (%)	99.9 (99.6)	99.9 (99.5)	99.9 (99.2)
Redundancy	7.1 (6.7)	7.1 (6.7)	7.1 (6.6)
⟨*I*/σ(*I*)⟩	9.6 (0.6)	13.0 (0.7)	14.1 (0.9)
*R*_meas_	0.110 (2.321)	0.080 (2.459)	0.084 (1.964)
CC_1/2_	0.998 (0.427)	0.999 (0.583)	0.999 (0.530)
Refinement	*Refmac*	*Refmac*	*Refmac*
Resolution range (Å)	50–2.19	50–2.02	50–2.27
*R*‐factor/free *R*‐factor (%)	19.9/21.9	21.8/23.9	19.8/22.1
No. of atoms	6399	6471	6438
No. of solvent atoms	142	216	140
Ramachandran distribution (% favored, allowed, outlier)	98.2, 1.8, 0	98.2, 1.8, 0	98.2, 1.8, 0
RMS bonds (Å), angles (°)	0.008, 1.74	0.008, 1.65	0.008, 1.72
Average *B* value (Å^2^)	61.9	58.1	64.9

**Fig. 3:**
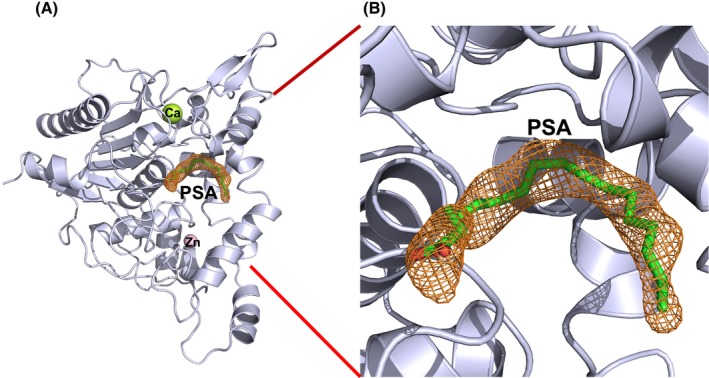
Crystal structure of the SAL‐PSA complex. (A) Schematic representation of the SAL structure. The PSA molecule is shown as sticks. Calcium and zinc ions are shown in green and pink, respectively. (B) Close‐up view. The PSA molecule is shown as a stick in green. The omitted map of PSA is also shown as an orange mesh contoured at 1.0 sigma. All molecular structure images were generated using *pymol* (http://www.pymol.org).

Using the crystal data set, the native structure was used as the model coordinates, and the molecular replacement method solved the structure of the SAL/PSA complex. The electron density maps of PSA with the same conformation for both A‐ and B‐chains were clearly obtained in the vicinity of the active site (Fig. 3A). The 3D structure was constructed by tracing PSA molecules onto this map, and the structure was further refined by adding water molecules and fatty acids. As a result, we determined a stereochemically correct structure with an *R‐*factor of 19.9% and an *R‐*free of 21.9% using 2.19 Å resolution data.

On the other hand, no electron density corresponding to the PSM was observed. Co‐crystallization was attempted with various types of other unsaturated fatty acids and their ester compounds: oleic acid and linoleic acid, which have the same length as PSA but different numbers and locations of double bond positions, and unsaturated fatty acids with different carbon chain numbers were also co‐crystallized and data collected. Analysis of various complex structures was also attempted for other unsaturated fatty acids, oleic acid, linoleic acid, elaidic acid, erucic acid, methyl cis‐11‐octadecenoate, methyl trans‐9‐octadecenoate (Fig. S2). However, no electron density maps were available for them either.

More than 10 data sets of PSAs were analyzed for the SAL‐PSA complex structures, including some at lower resolution than the data presented here, all of which formed a complex with SAL. The SAL‐S116A mutant also showed complex formation in more than 10 data sets. These results indicate that only the cis‐6 unsaturated type of PSA binds tightly to SAL and shows a strong electron density map at the active site (Fig. 3A,B).

### General structure and active site of SAL

**Fig. 4:**
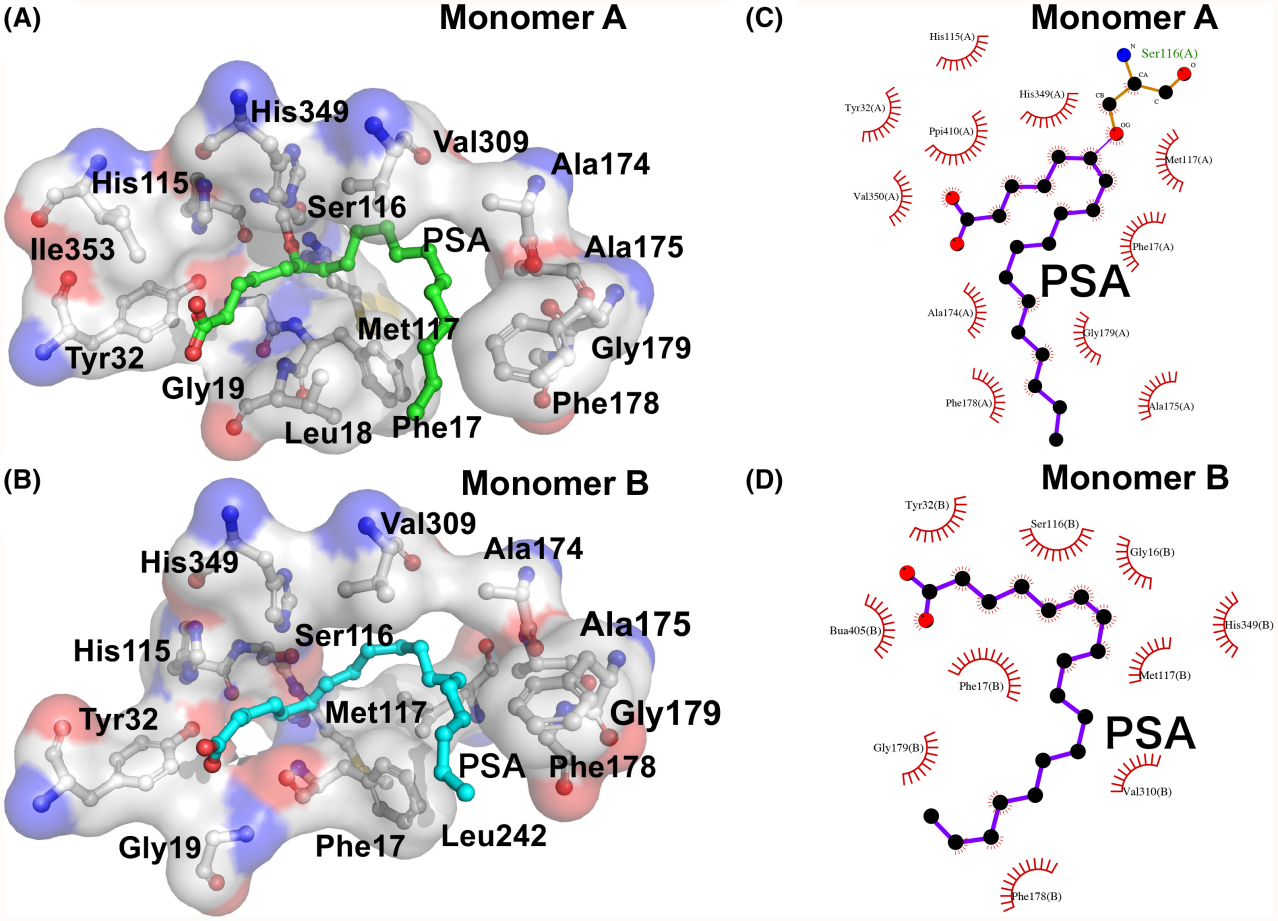
Binding interaction between SAL and PSA. (A) Close‐up view of the mode of PSA recognition at the catalytic site of the A‐chain of SAL. Residues indicating PSA recognition are highlighted as sticks and surface models. Positively charged regions are shown in blue and negatively charged regions are shown in red. The PSA molecule is shown as a green stick. Tyr32 is in contact with the carboxyl group of PSA. (B) The major interactions of PSA in the active site of the A‐chain of SAL are shown using a *LIGPLOT* ^+^ diagram [29]. (C) Close‐up view; the recognition mode of PSA at the catalytic site of the B‐chain of SAL; the PSA molecule is shown as a blue stick. (D) The main interaction of PSA at the active site of the B‐chain of SAL is shown using a *LIGPLOT* ^+^ diagram [29].

The monomeric structure of SAL was similar to that reported in previous studies [13]. It retained the typical α/β‐hydrolase fold consisting of 13 β‐strands, 13 α‐helices, and six 3_10_‐helices (Fig. 3A). Consistent with previous studies, SAL had two large hydrophobic pockets extending from the active center, Ser116, with hydrophobic alkyl chain fatty acids attached to these grooves [13] (Fig. 4A,C). The entrance to this groove was formed by a series of hydrophobic amino acids (Phe17, Leu18, Ty29, Pro30, Phe178, Met188, Phe286, Leu287, Val350, Val355). The active center, Ser116, was located in a shallow pocket 23 Å wide and about 11 Å deep (Fig. 4A) [13].

The nucleophilic atom Ser116 of the GXSXG lipase motif was located in the so‐called “β‐elbow” loop between the β‐5 chain and the α‐4 helix of the α/β‐hydrolase center [17]. Ser116 is very important for the reaction with the substrate and has the same catalytic mechanism as other serine hydrolases. The nitrogen atoms in the main chains of Phe17 and Met117 form oxyanion holes and can stabilize intermediate transition states during the reaction (Fig. 4A,B).

**Fig. 5:**
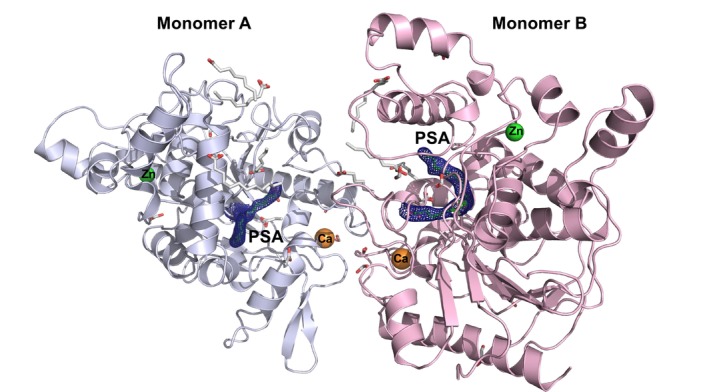
SAL dimer complexed with PSA and fatty acids. Each monomer is shown in blue (chain A) and pink (chain B); the PSAs bound to the A and B molecules are shown in the electron density map (calculated at 1.0 sigma) above, and the fatty acid fragment molecules are shown as green and white sticks respectively.

One molecule of zinc and calcium ions per monomer was bound to SAL (Figs 3A and 5), and the binding positions and modes were identical to previous studies [13].

As in previous studies, the SAL obtained by structural analysis formed a dimeric structure in the crystal (Fig. 5) [13]. It was already identified as a dimer in solution by gel filtration chromatography and SEC‐SAXS (Fig. S3). The dimer was formed by hydrophobic interactions involving 12 residues near Ca^2+^, with Asp281, Leu282, Phe286, Phe357, Leu358, and Phe360 forming a hydrophobic cluster in the loop region surrounding Ca^2+^ ions. One molecule of PSA was bound to each of the A‐ and B‐chains of the SAL dimer (Fig. 5).

### Structural determination of the orientation of PSA molecules

For the interpretation of the electron density map of the active site corresponding to PSA, two possible orientations of the PSA structure were considered depending on the arrangement of the carboxyl groups (Fig. S4a–c).

Petroselinic acid is an 18‐carbon cis compound with an unsaturated double bond from which the carbon chains flow in two directions, each in a cis conformation. Like other unsaturated fatty acid complexes, it has a junction‐like curved conformation from the position of the double bond (Fig. 1), with alkyl chains extending left and right in two directions. This molecule has a short carbon chain of 5 carbons with a carboxylic acid on one side and an alkyl chain consisting of a long carbon chain of 11 saturated carbons on the other side.

Structure determination of the PSA model molecule was performed automatically by combining real‐space refinement with *Coot* and *Refmac*. The maps obtained by the molecular replacement method allowed two interpretations of the long, curved, crescent‐shaped electron density in the vicinity of the active site.

The electron density extended from serine 116 in the active site and was covalently bound to the PSA molecule (Fig. S4a) The short and long electron densities were extended at the boundary there (Fig. S4a).

After analysis by the molecular replacement method, the covalent bond between PSA and Ser116 was observed to be linked only in molecule A, but was broken in molecule B in the data analyzed at 2.19 Å resolution. On the other hand, in the 2.27 Å resolution analysis, the electron density bond between the side chain of Ser116 and PSA was observed in both molecule A and B.

In the map corresponding to the active site PSA, neither end of the electron density had a distinct shape corresponding to a carboxylic acid (Fig. S4a–c). We traced the carboxyl group of the two electron densities in both directions away from Ser116 in two ways (Fig. S4b,c). One was constructed by tracing the molecule with a C1 carboxylic acid group at the short electron density end and fitting the molecule to the map using *Real Space refinement tools* (Fig. S4b), and the other was constructed by fitting the molecule to the map with a C18 alkyl chain carbon at the end (Fig. S4c). Each structure was then refined separately in *Refmac*. We were able to approach a more reasonable interpretation by comparing the two structures (Fig. S4b,c).

In determining the structure, we also referenced the structure of a previous bacterial lipase, *Thermomyces* (*Humicola*) *lanuginose* lipase (hereinafter abbreviated as TLL) complexed with oleic acid (hereafter referred to as OA) (PDB ID: 1GT6) (Fig. S5) [18]. Like PSA, OA is an unsaturated fatty acid with 18 monoatomic elements, but the position of the double bond differs from that of PSA [18]. PSA has a double bond between the sixth and seventh carbons of the carboxylic acid, whereas OA has a double bond between the ninth and tenth carbons (Fig. S2). In this complex structure, the OA molecule had two completely different positional binding modes: one was the A‐molecule mode, in which the carboxyl group of OA was bound directly near the active site [18]. In this structure, the overall electron density was unclear and unconfirmed [18]. More clearly visible was the other B molecule, a structure in which the double bond portion of the OA (C9‐C10) was located near the S146A residue of the catalytic group of the active site (which is a serine in complex with the inactive mutant) (Fig. S5). However, even in the analysis of the TLL/OA complex, the oxygen on one side of the carboxylic acid moiety was not very clearly visible [18]. The reason for this was discussed in the paper as the mobility of the oleic acid molecule [18].

In the PSA structure determination, the double bond position did not interact at all with the SAL in the molecule with the short electron density end traced to the alkyl group. The difference Fourier map after refinement showed a large positively charged peak near Ser116 and at the alkyl end, suggesting that this end may be a carboxyl group rather than an alkyl chain and that the trace was probably reversed (Fig. S4c).

At 2.27 Å resolution, the covalent SAL/PSA complexes show a clear linkage between the C6 carbon of the PSA double bond and the electron density of serine 116 for both A and B molecules, and the covalent bond is clearly visible in the maps. The double bond moiety was also bound to the electron density extending from the active site oxyanion hole consisting of the main chain N atom of Phe17 and 17 of SAL and the main chain N atom of Met117 (Fig. S4d).

These results allow us to locate the double bond and suggest the certainty of a structure that follows the carboxylic acid to the end, with the shorter electron density extending from Ser116 (Fig. S4d). Regarding the electron density of the carboxylic acid, the carboxylic acid terminus of PSA is close to and interacts with the Tyr32 residue, although one of the oxygens is not well visible (Fig. S4c).

In PSA, Ser116 was attached to the sixth carbon at position C6, counting from the carboxyl group (Fig. S4d). In OA, the mutant had alanine instead of serine, but Ala146 was located near the carbon of the double bond at the C9 position (Fig. S5).

*Staphylococcus aureus* lipase has a large hydrophobic pocket, and PSA bound across the hydrophobic pocket extending in two directions from the active site of Ser116. PSA with long alkyl chains adopted an arcuate caterpillar structure along this hydrophobic pocket of SAL, with the terminal carboxylic acid moiety interacting with the Tyr32 residue (Fig. 4A–D). The PSA was sandwiched between the hydrophobic pockets from the top and bottom, and all PSA molecules appeared to fit into the pockets. The carboxylic acid terminus of PSA, Tyr32, Ile353, and His115 residues were trapped in a hydrophobic cluster of Leu18 and Phe17 residues. The long alkyl chain then interacted with His348, Val309, Ala174, Ala175, and Phe178, trapping the alkyl terminus and, in a roundabout way, by the hydrophobic cluster of Leu18 and Phe17 residues (Fig. 4A–D).

### Interaction between SAL and PSA

**Fig. 6:**
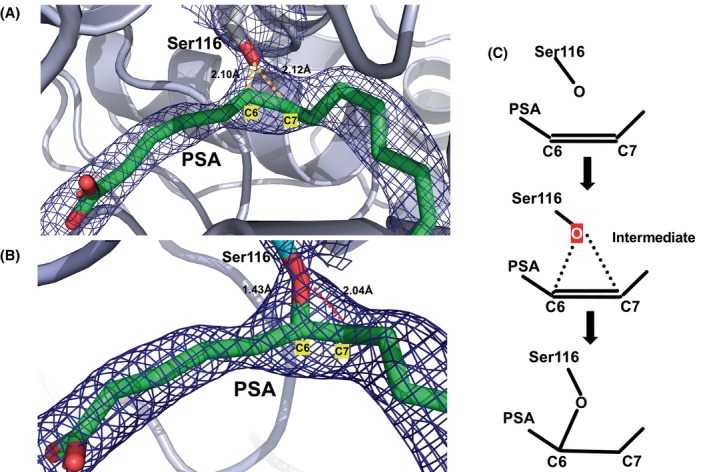
(A) Close‐up of the 2Fo‐Fc electron density map of the SAL catalytic site. Electron density maps calculated at 1.0 sigma are shown in the blue mesh, and the distance between the O atom and the C6 and C7 carbons of Ser116 is shown numerically and indicated by the orange dotted line. An intermediate structure was observed, with the oxygen of the active serine approaching the position of the two carbons of the double bond. (B) The 2Fo‐Fc electron density map of the SAL catalytic site in the covalent complex is shown. The oxygen atom (red) of Ser116 (blue) was covalently bonded to the C6 carbon of PSA (green) at a distance of 1.43 Å. (C) A schematic of the PSA binding pattern is shown: nucleophilic attack by the oxygen at Ser116 of PSA binds to the C6 carbon of PSA. These structures allowed snapshots of SAL/PSA near the reaction intermediate (A) and after reaction (B).

One molecule of PSA was bound to each of the A‐ and B‐chains of the SAL dimer (Fig. 5). Interestingly, PSA was bound to almost the same position in the A‐ and B‐chains, but the binding mode was different near the active Ser116 (Fig. 4A–D). In the A‐chain, the carbons at the C6 and C7 positions, counted from the carbon atom of the carboxylic acid at the PSA terminus, form a double bond, and the oxygen atom of Ser116 is located near the center of this double bond, forming a covalent structure with the C6 carbon atom (Fig. 6A,B). The distances between the oxygen atom and the C6 and C7 carbons of the side chain of the catalytic residue Ser116 were 2.10 and 2.12 Å, respectively, and were slightly closer to the carboxylic acid side (Fig. 6A). However, PSA molecules on the other B‐chain of the SAL dimer showed no interaction with Ser116, and the distance between the oxygen atom of the Ser116 side chain and the C6 carbon was as large as 3.14 Å (Fig. 4C,D).

For the covalent complexes, we combined real‐space refinement on the *Coot* with refinement on the *Refmac* to eliminate human intervention and automatically determine the structures. The electron density map of the bond from Ser116 to PSA was clearly visible before refinement for both A and B. The distance between the oxygen atom of the Ser116 side chain and the C6 carbon was 1.43 and 1.41 Å for the A and B molecules, respectively, and the distance to the C7 carbon was 2.04 and 1.93 Å for the A and B molecules, respectively.

These results allowed us to capture the conformation of the SAL/PSA complex in both the PSA‐bound state and in the state where SAL initiates the reaction. We found a conformational mode of the active serine reaction intermediate state (Fig. 6A–C), suggesting that the covalent conformation between Ser116 and the C6‐C7 double bond of PSA may capture a snapshot near the intermediate state where the reaction process is initiated (Fig. 6A–C). The observation of this conformational state suggests that unsaturated fatty acids bind to the active serine double bond and inhibit the enzymatic reaction.

Although this mode of binding of PSA to SAL is not presumed to be irreversible based on the experimental results using the Econo Pac 10 Dg column (Bio‐Rad, Hercules, CA, USA) (Fig. S6). SAL‐PSA solution B, in which SAL inhibited an excess amount of PSA, was applied to the 10 DG column, and then solution C, in which PSA was removed, the inhibition state was almost equivalent to that of the solution to which an excess amount was added (Fig. S6a,b). Compared to SAL solution A without inhibition, there was some residual activity, but the majority of the enzymes remained inhibited (Fig. S6a,b). This suggests that PSA may form a strong covalent bond with SAL.

### Crystal structure of the inactive mutant S116A‐SAL in complex with PSA

**Fig. 7:**
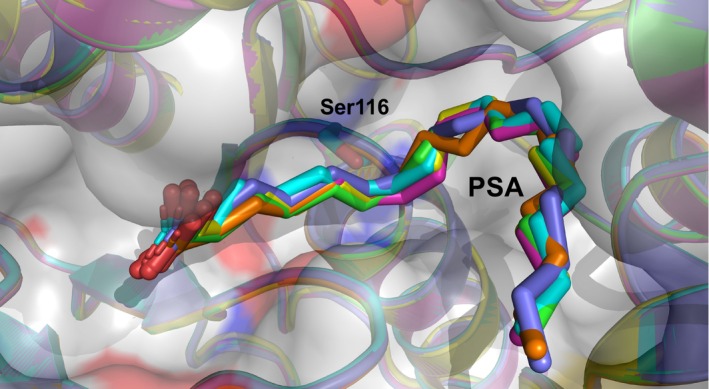
Superimposed structures of wild‐type SAL and the S116A mutant complex with PSA in the monomer. The A and B molecules of the wild‐type SAL, the S116A mutant, and the covalent complex are shown in cartoon models highlighted in green, cyan, magenta, yellow, blue, and orange, respectively. The surface model is added at 50% transparency. PSA and Ser116 are shown as sticks.

The S116A mutant, in which Ser116, the active catalytic residue of SAL, was replaced by Ala, was crystallized in a complex with PSA. Interestingly, the S116A/PSA mutant crystal could be analyzed at 2.02 Å resolution and contains one molecule of PSA each on both the A‐ and B‐chains (Table 1). The conformation of PSA was almost identical to that of the wild‐type complex, except for the absence of the Ser116 side chain (Fig. 7). It was bound in almost the same position as the B molecule of the SAL/PSA complex (Fig. 7).

The structures of all six obtained PSA molecules were similar, with the double bond located in the oxyanion hole near the active Ser116 (Fig. 7). As seen in the covalent bond complex, the carbon atom on the double bond side with the carboxyl group is susceptible to nucleophilic attack from the oxygen atom of Ser116. Of the two carbons on the double bond, the carbon on the side where the carboxyl group is attached is more positively charged by the electronegative group. Therefore, the oxygen atom of the nucleophile Ser116 could attack the carbon at the C6 position.

In this study, three types of PDB data have been deposited: The first is a complex structure of an inactive mutant of S116A with PSA (PDB ID: 8K7Q). The second is the highest resolution structure of wild‐type SAL complexed with PSA, in which only one of the two molecules of PSA is nearly covalently bound to Ser1116 of SAL (PDB ID: 8K7P). The other is a SAL/PSA complex in which both AB molecules are covalently bound to SAL (PDB ID: 8YIB). From these three structures, we believe we have captured three steps: the pre‐reaction structure, the intermediate structure near the transition state, and the covalently bound structure after the reaction (Fig. 6A–C).

### Structure–activity relationship of SAL‐PSA

Co‐crystals were also obtained for PSM, in which a methyl group was substituted for the oxygen of the carboxylic acid of PSA (Fig. 1), and diffraction data sets could be collected. However, the analysis did not yield the electron density corresponding to PSM, and the bonding state between PSM and SAL could not be confirmed. This result was attributed to the introduction of a methyl group into the carboxylic acid, which bulks up the binding site and reduces the interaction with Tyr32 (Fig. 4A–D).

The IC_50_ concentration against SAL in the activity assay was 3.4 μm for PSA (Fig. 2). In contrast, it was 10.5 μm for PSM, more than three times weaker (Figs 1 and 2). This may be due to the introduction of a methyl group into the carboxylic acid, which reduced the interaction with the Tyr32 residue of SAL in terms of both charge and bulk.

Co‐crystals were also prepared and structurally analyzed for other long‐chain alkyl unsaturated fatty acids and their esters, such as cis‐9, cis‐11, and cis‐13, but only PSA of the cis‐6 type was able to obtain electron density in the active site (Fig. S2).

### Predicted structure of PSA binding to gastric lipase

Previously, we confirmed that the anti‐obesity drug inhibitor orlistat interacts strongly and irreversibly with SAL [13]. From structural comparisons with the mammalian dog gastric lipase, we concluded that it binds to the human‐type gastric lipase with similar effect.

The dog gastric lipase (DGL; PBD ID: 1K8Q) had an open structure, whereas the human gastric lipase (HGL; PBD ID: 1HLG) had a closed structure with a lid domain [13]. The structure entered in the PDB was in closed form, with the active site covered by a lid domain. When the *Alpha Fold2* was used to predict the structure of HGL, it was possible to predict the structure of the open form, in which the active site is open.

**Fig. 8:**
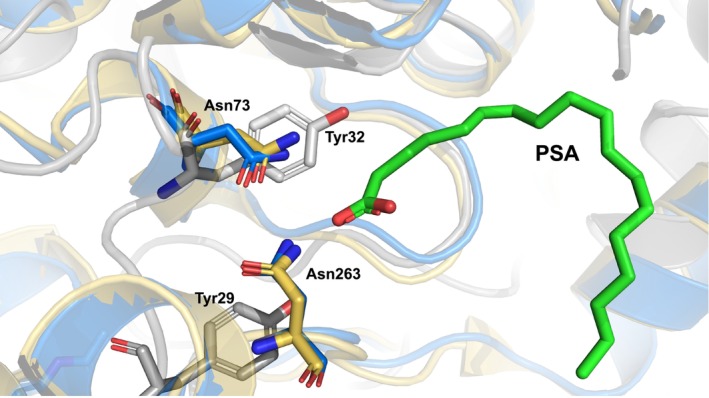
Predicted molecular docking of PSA in SAL, DGL and HGL. The three lipases are superimposed and the amino acid side chains that can interact with the carboxyl terminus of PSA are indicated by sticks. White is SAL, gold is DGL and blue is HGL.

The structure‐predicted binding mode of PSA to human‐type gastric lipase was consistent with binding to the oxyanion hole, where two Asn73 and Asn253, but not Tyr residues were present in the vicinity of the carboxyl terminus of PSA (Fig. 8). These two asparagine residues were located where they could electrostatically interact with the carboxyl terminus of the PSA (Fig. 8). Therefore, a possible interaction between the carboxyl terminus of PSA and these asparagine residues is suggested.

Based on these structural predictions, the binding of PSA to human gastric lipase was estimated, suggesting that PSA, an unsaturated fatty acid derived from parsley oil, may inhibit human gastric lipase. If PSA from parsley oil can inhibit the activity of human gastric lipase, it is expected to be a new type of anti‐obesity drug. There have also been suggestions that parsley may be a food with strong weight loss potential. Therefore, it has been suggested that PSA is a compound with dual functions: antimicrobial and anti‐obesity effect.

## Conclusions

In conclusion, PSA, a long‐chain alkyl fatty acid that inhibits *Staphylococcus aureus* biofilm formation, also inhibits SAL activity, with an IC_50_ of 3.4 μm. The crystal structures of PSA complexed with wild‐type SAL and the inactive mutant S116A‐SAL were determined, respectively. The results showed that PSA interacts with SAL by inserting its double binding site into an oxyanion hole near the active site of SAL. The active conformation also showed a covalent interaction with Ser116, a catalytic residue of one of the molecules in the A‐ and B‐chains.

It was found that unsaturated fatty acids of the cis‐6 type, which have a double bond between positions 6 and 7 counting from the carboxylic acid terminal carbon of PSA, are effective inhibitors and that this double bond must fit exactly into the oxyanion hole of SAL. It was also suggested that the lack of esterification of the terminal carboxylic acid of the unsaturated fatty acid is important for inhibitory activity.

Based on this structural information, the predicted structures of the mammalian‐type gastric lipase and PSA suggest that PSA may bind to the human‐type gastric lipase. Thus, PSA has shown the potential not only for antimicrobial effects but also for anti‐obesity effects.

## Materials and methods

### Expression and purification of *pColdII‐SAL*

As previously reported, PCR was performed using oligonucleotide primers 5′‐ACGCCATATCGCCGAAAGG‐3′ and 5′‐GGCAGGGATCTTAGATTCTG‐3′ with *pColdII‐SAL* as the template PCR products were incubated with DpnI (Toyobo, Osaka, Japan) and the template DNA was digested and then transformed into DH5α‐competent cells (Toyobo). The amplified plasmid was purified, and the mutanted nucleotides were identified. The plasmid was introduced into *E. coli* strain BL21 (DE3) (Agilent, Santa Clara, CA, USA), cultured and stored as glycerol stock.

A glycerol stock containing the recombinant SAL gene in the *pColdII v*ector of Escherichia coli, harbored by BL21 (DE3), was precultured with 40 mL LB medium containing 50 μg·mL^−1^ ampicillin at 37 °C in 200 mL Meyer flasks [13, 19]. Ten milliliters of this preculture solution was transferred to a 1 L solution of LB medium containing ampicillin, and 0.1 mm IPTG was added to induce protein expression, followed by incubation at 15 °C for 24 h. The bacteria were collected, crushed, filtered, and purified by nickel affinity chromatography and two types of ion exchange chromatography; approximately 50 mg of highly purified SAL was obtained from 1 L of medium, which showed a single band on SDS electrophoresis analysis.

As previously reported, the cells were then collected by centrifugation at 277 K for 30 min at 2500 ***g***, washed with TE buffer, and suspended in lysis buffer (50 mm Tris–HCl buffer pH 8.0 containing 0.3 m NaCl and 10 mm imidazole). The suspension was sonicated at 273 K using an Ultrasonic Disruptor UD‐211 (Takara Tomy, Tokyo, Japan), centrifuged and the supernatant was filtered and sterilized. The supernatant was purified by immobilized metal affinity chromatography (Nuvia IMAC resin, BIO‐RAD). Proteins were eluted with an imidazole concentration gradient (10–400 mm); the SAL fraction was collected and dialyzed in buffer (0.2 m NaCl 10 mm MES pH 6.5) to remove imidazole. Further purification was performed by SP (Toyopearl) column chromatography, loaded with dialyzed proteins and eluted with a NaCl concentration gradient (0.2–1.0 m). The fractions containing SAL proteins were combined and dialyzed in 0.2 m NaCl 10 mm MES pH 6.0. The dialyzed SAL solution was then subjected to MonoQ column chromatography (GE Healthcare, Chicago, IL, USA) to remove foreign material, and the flow‐through fraction was collected as the final purified protein (Fig. 1).

### Crystallization

The composition of the crystallization sample buffer was 10 mm MES pH 6.0, 0.1 m NaCl, and the protein concentration was concentrated to approximately 30 mg·mL^−1^ by ultrafiltration using Vivaspin15 for crystallization. Protein concentration was estimated by the Bio‐Rad protein assay kit (Bio‐Rad Laboratories) with γ‐globulin as the standard protein.

The concentrations of various inhibitors were adjusted to 5–10‐fold molar equivalents or greater relative to the concentration of SAL. Concentrated SAL was mixed with a 5–10 fold molar excess of inhibitor dissolved in DMSO and incubated overnight.

The sitting drop vapor diffusion method was performed at 295 K using a Cryschem plate; 500 μL of crystallization solution (reservoir) was added to the bottom, and 2 μL each of the mixture of crystallization solution and protein solution was dropped into the top and sealed [19].

### Data collection, structure determination, and refinement

X‐ray diffraction data sets were collected at 100 K in a nitrogen gas stream using a mother solution with 30% glycerol added as a cryoprotectant at SPring‐8 beamlines BL41XU and BL44XU. The X‐ray wavelength was adjusted to 0.9 Å, the oscillation angle was 0.1°, and a total of 180° images were collected. The distance from the crystal to the detector was 300 mm. The collected diffraction images were integrated and scaled with the *XDS* program package [20] using the *KAMO* automatic program system [21] at SPring‐8. The data set was taken out at the limit of resolution, where the value of CC 1/2 is about 0.5 [22].

The molecular replacement method was used for structure determination with the program *Phaser* in the *CCP4* package [23, 24]. The native structure of SAL (PDB ID: 6KSI), already determined in a previous study, was used as a search model [13]. The *COOT* program was used to build the PSA molecule and fit the molecular model [25], and *REFMAC5* was used to refine the structure [26].

To fit the model with electron density maps, the real‐space refinement tool of Coot [25] was used. The refinement process was carried out until convergence using Refmac [26].

The stereochemistry of the model molecules was modified based on the Ramachandran plot.

### Measurement of IC_50_ half‐inhibition values

As previously reported, enzymatic activity was determined by absorbance at 405 nm by degradation of *p*‐nitrophenyl butyrate (*p*NPB) ester substrate [12, 13, 27]. SAL and *p*NPB concentrations were adjusted to 0.002 and 0.08 mm, respectively. Each assay was performed in a 1 mL cuvette containing 50 mm HEPES (pH 7.5) with 10% DMSO at 25 °C. Different concentrations of inhibitors (0–1 mm) were prepared to obtain IC_50_ values. Before starting the SAL/*p*NPB reaction, SAL was pre‐incubated with inhibitors for 10 min. Substrate was then added and assayed at 25 °C. The absorbance of free *p*‐nitrophenol was continuously monitored at a wavelength of 405 nm, and the relative specific activity of SAL was calculated from the initial linear velocity. Inhibition experiments were performed by adjusting solutions of different concentrations that inhibited SAL activity by more or < 50%. Measurements were performed at least three times, and IC_50_ values were determined using data averaged at each concentration.

### Molecular docking of PSAs into DGL and HGL

The structure prediction of HGL (Fig. 8) was performed using *AlphaFold2* [28]. Structural refinement and energy minimization of the DGL‐PSA and HGL‐PSA complexes were performed using the *YASARA* Energy Minimization Server (http://www.yasara.org/minimizationserver.htm).

## Conflict of interest

The authors declare no conflict of interest.

### Peer review

The peer review history for this article is available at https://www.webofscience.com/api/gateway/wos/peer‐review/10.1002/2211‐5463.13808.

## Author contributions

KK designed the research. JK, SK, YO, TH, MY, TH, and KK performed the research. JK, SK, YO, TH, MY, TH, and KK analyzed the data. JK, KK, and SK coordinated the work and drafted the manuscript.

## Supporting information


**Fig. S1.** A SAL/PSA complex crystal obtained from the co‐crystallization experiments.
**Fig. S2.** List of chemical structural formulas of unsaturated fatty acids tried for co‐crystallization in this study.
**Fig. S3.** SEC (Size Exclusion Chromatography)‐SAXS elution profile.
**Fig. S4.** Figure showing details of modeling the PSA molecule.
**Fig. S5.** Superimposed view of the structure of the SAL/PSA molecular complex and the TLL/OA complex.
**Fig. S6.** PSA inhibition studies.

## Data Availability

All data supporting the findings of this study are available within the paper and its Supporting Information. Additional requests can be obtained from the corresponding author: kengo@kit.ac.jp.
