# Supplementary material for: Crystal structure of Staphylococcus aureus lipase complex with unsaturated petroselinic acid
Source: FEBS Open Bio. 2024 May 16;14(6):942–54. doi: 10.1002/2211-5463.13808 (PMC11148114; doi:10.1002/2211-5463.13808)
Supplement: Supplementary file 1 — Fig. S1. A SAL/PSA complex crystal obtained from the co‐crystallization experiments. Fig. S2. List of chemical structural formulas of unsaturated fatty acids tried for co‐crystallization in this study. Fig. S3. SEC (Size Exclusion Chromatography)‐SAXS elution profile. Fig. S4. Figure showing details of modeling the PSA molecule. Fig. S5. Superimposed view of the structure of the SAL/PSA molecular complex and the TLL/OA complex. Fig. S6. PSA inhibition studies. [file FEB4-14-942-s001.docx]

**Supplemental figure**

**Fig. S. 1**

**Fig. S. 1**. A SAL/PSA complex crystal obtained from the co-crystallization experiments. The crystal had approximate maximum dimensions of 0.5 x 0.4 x 0.4 mm.

**Fig. S. 2.**

**Fig. S. 2. List of chemical structural formulas of unsaturated fatty acids tried for co-crystallization in this study.** Cis-6 means that the double bond in the cis conformation appears in the sixth position counting from the carboxyl terminal carbon.

**Fig. S. 3.**

**Fig. S. 3**. **SEC (Size Exclusion Chromatography)-SAXS elution profile.**

First half peak: Guinier radius 52 Å, estimated molecular weight 600 kDa. Middle peak: Guinier radius 34Å, estimated molecular weight 85-95kDa (dimer) ⇒ SAXS analysis attempted with scattering data in the yellow-green region The single peak shows the dimeric form of SAL.

**Fig. S. 4.**

**Fig. S. 4 Figure showing details of modeling the PSA molecule.** (a) From the catalytic residue Ser116, the 2FoFc map was split in two directions. In the figure, short chains were observed on the left and long chains on the right. The PSA molecule was positioned based on the electron density and refined using the real space refinement tool. Refinement calculations were subsequently repeated in *Refmac* until the structure was stabilized. (b) When the carboxylic acid terminus was placed on a short chain, the entire molecule fit exactly and there were few discrepancies in the difference Fourier map. (c) Refinement results for the carboxylic acid terminus on a long chain. The difference Fourier map revealed a large positively charged peak (green) at the alkyl end, indicating that this part is a carboxylic acid. (d) The electron density map near the active site and the final model of the PSA molecule are displayed in the figure. The refined 2Fo-Fc map displays electron density extending from three directions towards the PSA double bond. The electron density was connected from the nitrogen atoms of Phe17 and Met117 (blue), which are known as oxyanion holes, and the oxygen atom of Ser116 (red). These electron density connections confirm the certainty of the PSA molecular structure.

**Fig. S. 5.**

**Fig. S. 5. Superimposed view of the structure of the SAL/PSA molecular complex and the TLL/OA complex.** The SAL/PSA complex ( shown in green) had Ser116 bound to the C6 carbon of PSA. In contrast, the TLL/OA complex ( blue) had the inactive mutant Ala146 located near the OA double bond.

**Fig. S. 6.**

**Fig. S. 6**  **PSA inhibition studies.**

SAL was inhibited with excess molar PSA (final 2 mM); then, unbound PSA was removed with a 10 DG column (Bio-Rad) and equilibrated with 10 mM Tris-HCl buffer (pH 8.0) supplemented with 0.2 M NaCl. SAL protein solutions were analyzed by SDS-PAGE before and after elution from 10 DG column. Residual activities of SAL were then detected by *p*NPB hydrolysis. Sample A is native SAL for control, sample B before elution and sample C after elution with 10 DG column.
